# Supplementary figures and images for: Nerve recovery from treatment with a vascularized nerve graft compared to an autologous non-vascularized nerve graft in animal models: A systematic review and meta-analysis
Source: PLoS One. 2021 Dec 2;16(12):e0252250. doi: 10.1371/journal.pone.0252250 (PMC8638852; doi:10.1371/journal.pone.0252250)

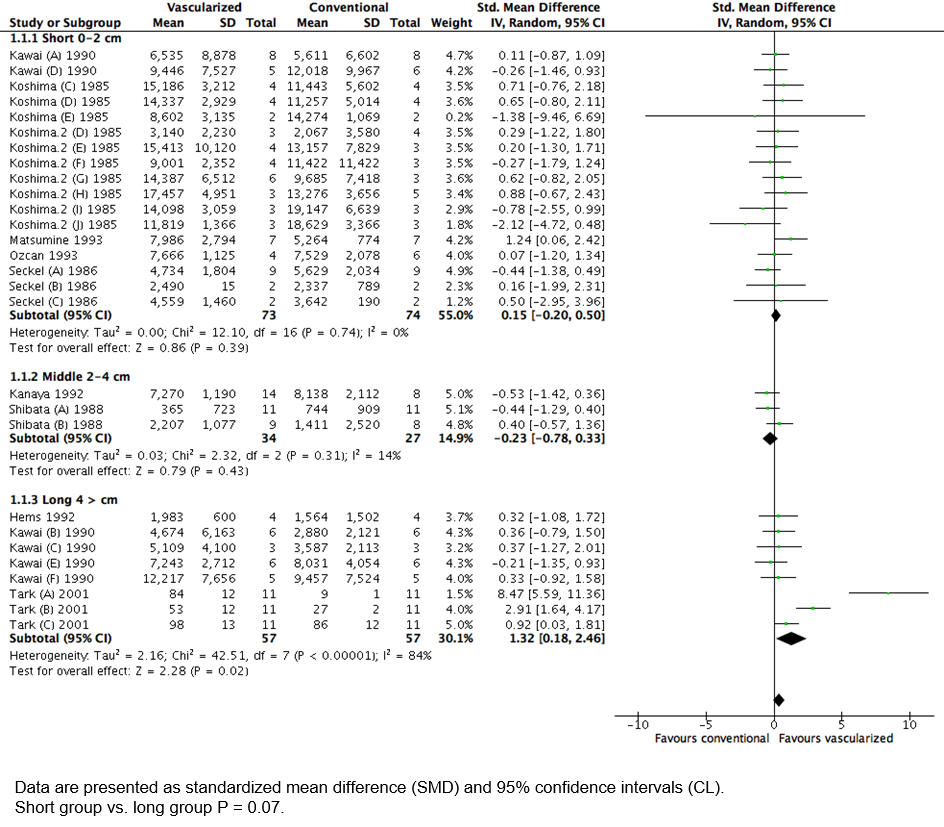

Supplement: S1 Fig — Data are presented as standardized mean difference (SMD) and 95% confidence intervals (CL). Short group vs. long group P = 0.07. (TIF) [file pone.0252250.s001.tif]

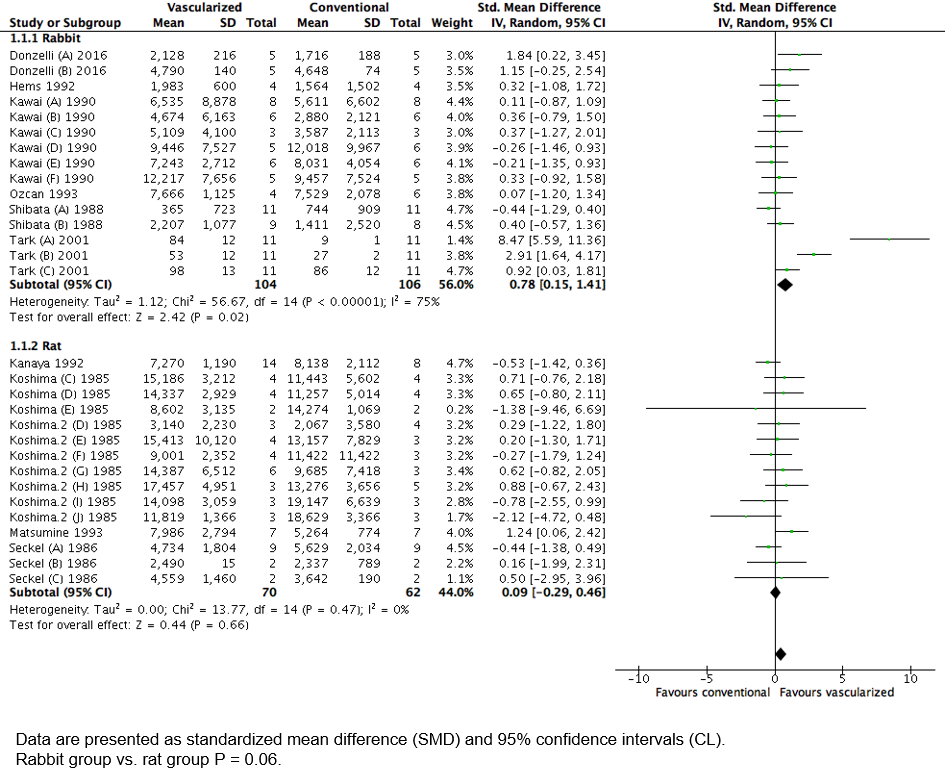

Supplement: S2 Fig — Data are presented as standardized mean difference (SMD) and 95% confidence intervals (CL). Rabbit group vs. rat group P = 0.06. (TIF) [file pone.0252250.s002.tif]

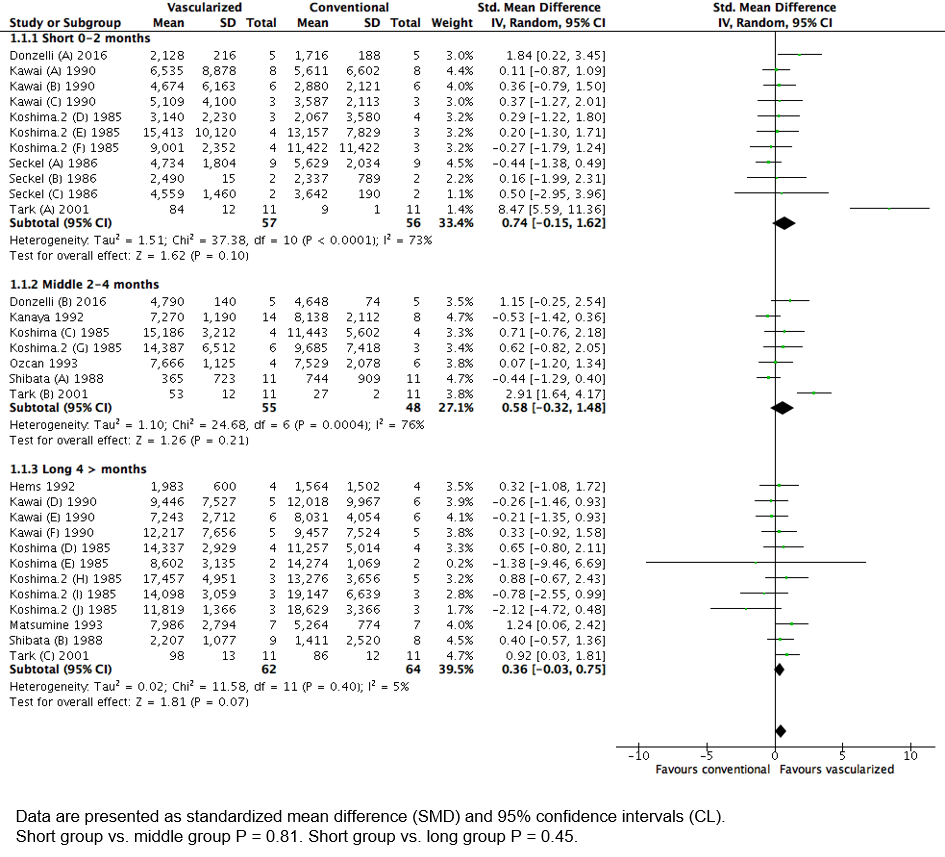

Supplement: S3 Fig — Data are presented as standardized mean difference (SMD) and 95% confidence intervals (CL). Short group vs. middle group P = 0.81. short group vs. long group P = 0.45. (TIF) [file pone.0252250.s003.tif]

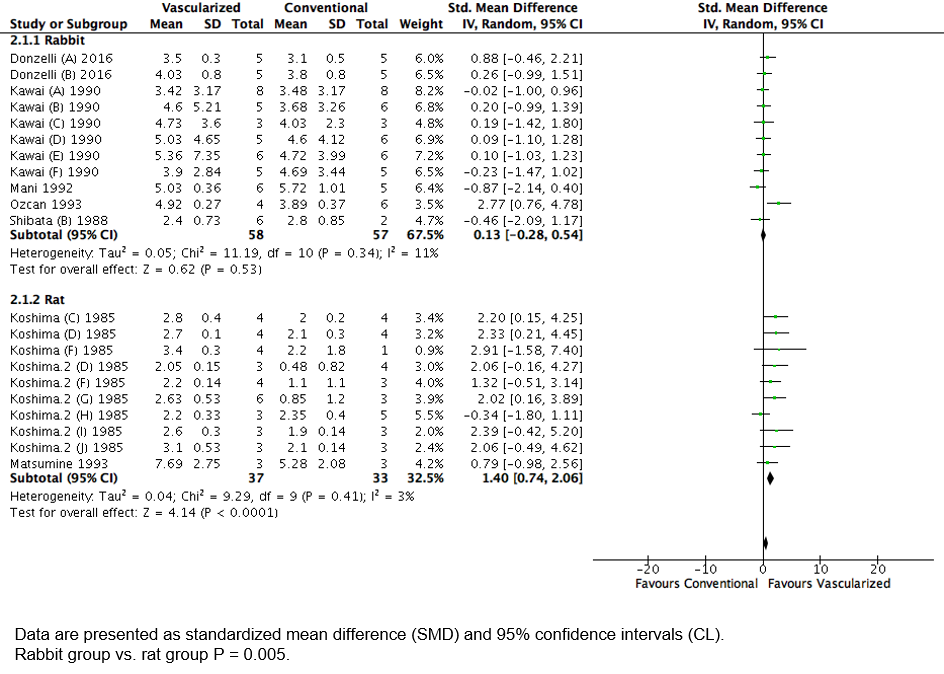

Supplement: S4 Fig — Data are presented as standardized mean difference (SMD) and 95% confidence intervals (CL). Rabbit group vs. rat group P = 0.005. (TIF) [file pone.0252250.s004.tif]

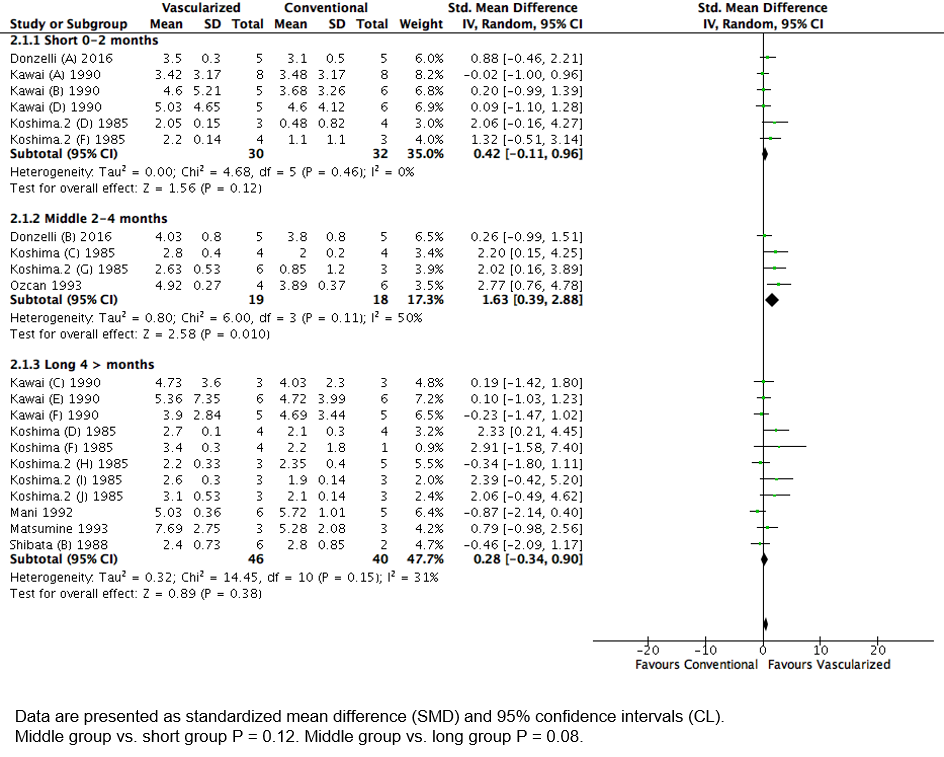

Supplement: S5 Fig — Data are presented as standardized mean difference (SMD) and 95% confidence intervals (CL). Middle group vs. short group P = 0.12. Middle group vs. long group P = 0.08. (TIF) [file pone.0252250.s005.tif]

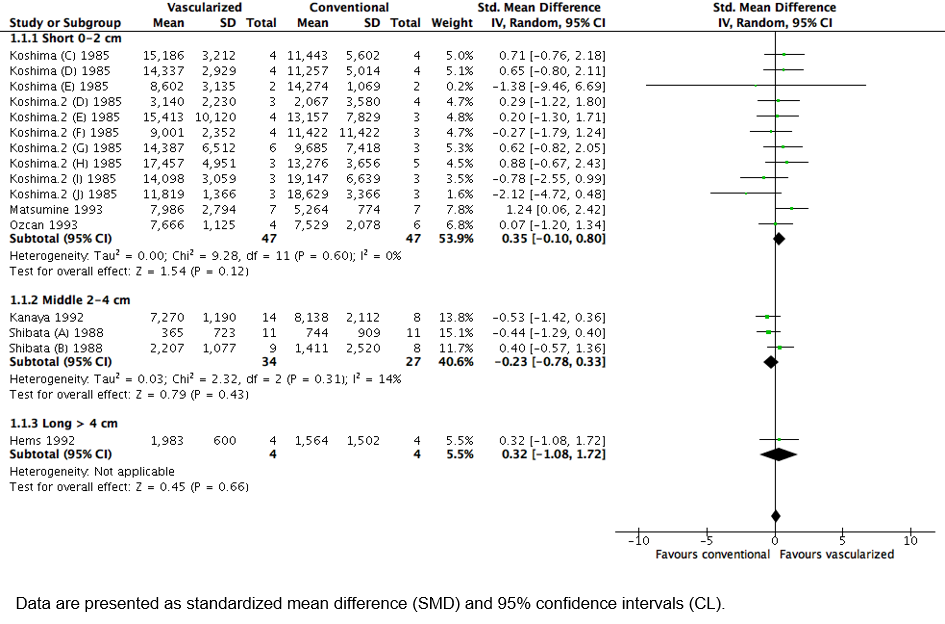

Supplement: S6 Fig — Data are presented as standardized mean difference (SMD) and 95% confidence intervals (CL). (TIF) [file pone.0252250.s006.tif]

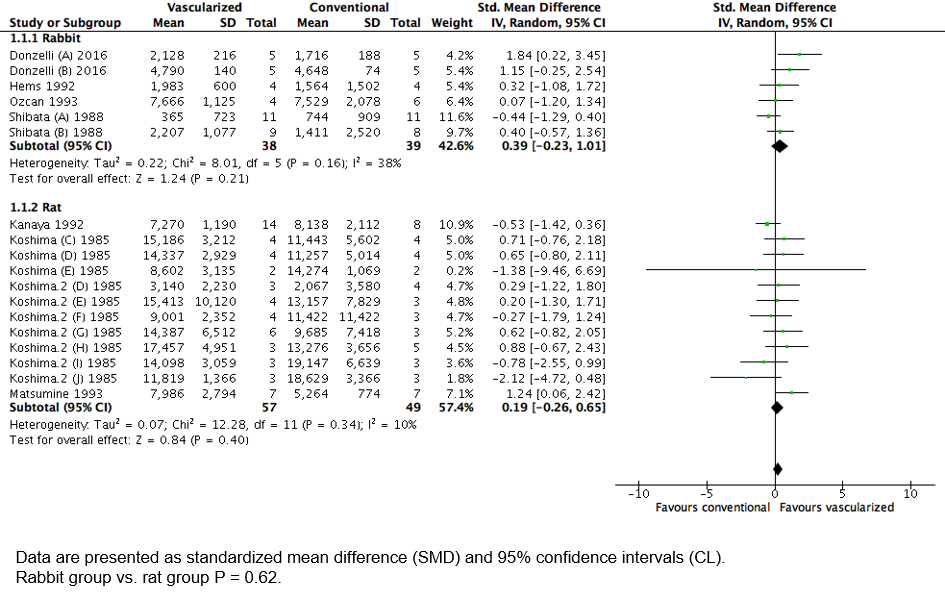

Supplement: S7 Fig — Data are presented as standardized mean difference (SMD) and 95% confidence intervals (CL). Rabbit group vs. rat group P = 0.62. (TIF) [file pone.0252250.s007.tif]

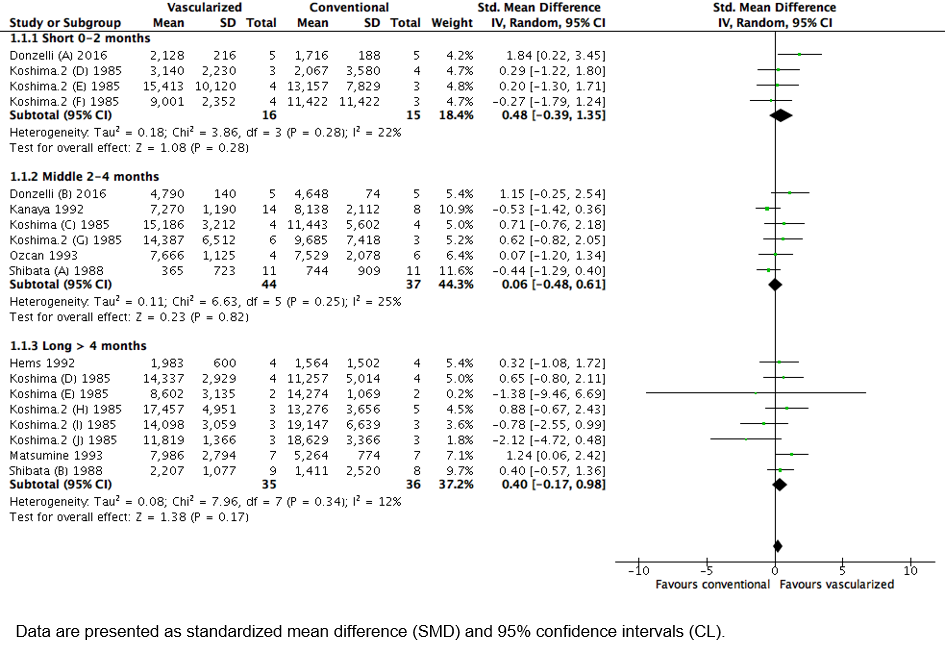

Supplement: S8 Fig — Data are presented as standardized mean difference (SMD) and 95% confidence intervals (CL). (TIF) [file pone.0252250.s008.tif]

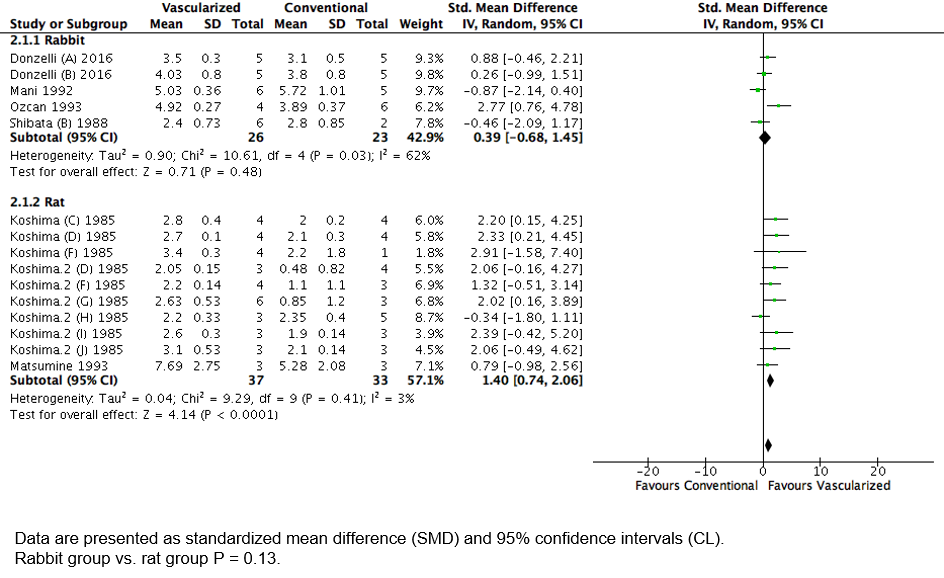

Supplement: S9 Fig — Data are presented as standardized mean difference (SMD) and 95% confidence intervals (CL). Rabbit group vs. rat group P = 0.13. (TIF) [file pone.0252250.s009.tif]

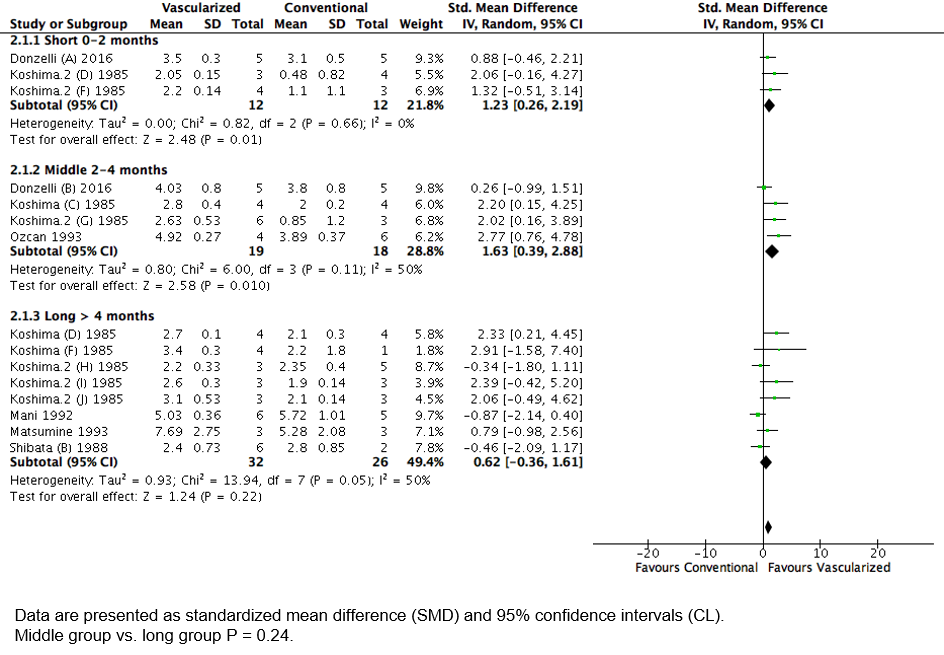

Supplement: S10 Fig — Data are presented as standardized mean difference (SMD) and 95% confidence intervals (CL). Middle group vs. long group P = 0.24. (TIF) [file pone.0252250.s010.tif]

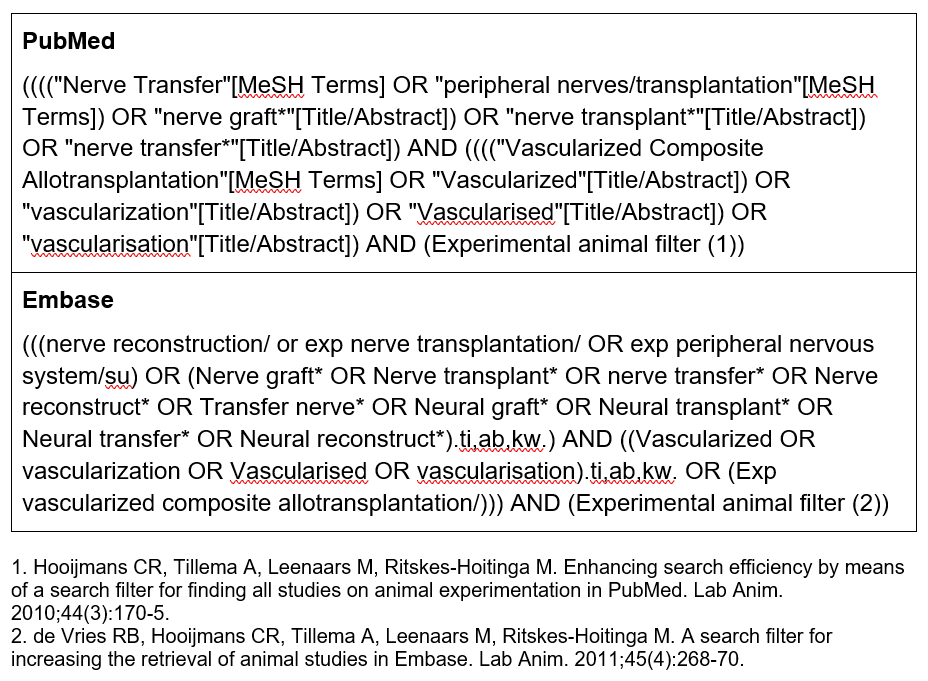

Supplement: S1 Table — (TIFF) [file pone.0252250.s012.tiff]
