# Supplementary material for: Nerve recovery from treatment with a vascularized nerve graft compared to an autologous non-vascularized nerve graft in animal models: A systematic review and meta-analysis
Source: PLoS One. 2021 Dec 2;16(12):e0252250. doi: 10.1371/journal.pone.0252250 (PMC8638852; doi:10.1371/journal.pone.0252250)
Supplement: S11 Fig — (PDF) [file pone.0252250.s011.pdf]

Raw data

Axonal count

study name	vng mean	vng sd	vng n	cng mean	cng sd	cng n	days	graft size (mm)
Donzelli et al., 2016 (A)	2128	216	5	1716	188	5	30	
Donzelli et al., 2016 (B)	4790	140	5	4648	74	5	90	
Hems et al., 1992	1983	600	4	1564	1502	4	250	50
Kanaya et al., 1992	7270	1190	14	8138	2112	8	84	25
Kawai et al., 1990 (A)	6535	8878	8	5611	6602	8	56	20
Kawai et al., 1990 (B)	4674	6163	6	2880	2121	6	56	40
Kawai et al., 1990 (C)	5109	4100	3	3587	2113	3	56	60
Kawai et al., 1990 (D)	9446	7527	5	12018	9967	6	168	20
Kawai et al., 1990 (E	7243	2712	6	8031	4054	6	168	40
Kawai et al., 1990 (F)	12217	7656	5	9457	7524	5	168	60
Koshima et al., 1985 (A)	0	0	4	0	0	4	28	15
Koshima et al., 1985 (B)	5655	5655	3	0	5655	3	56	15
Koshima et al., 1985 (C)	15186	3212	4	11443	5602	4	84	15
Koshima et al., 1985 (D)	14337	2929	4	11257	5014	4	112	15
Koshima et al., 1985 (E)	8602	3135	2	14274	1069	2	140	15
Koshima et al., 1985 (F)	11549	5097	4	-	-	-	168	15
Koshima et al.2, 1985 (A)	0	0	3	0	0	4	21	15
Koshima et al.2, 1985 (B)	4620	6534	3	0	6534	3	28	15
Koshima et al.2, 1985 (C)	1433	2027	3	0	2027	4	35	15
Koshima et al.2, 1985 (D)	3140	2230	3	2067	3580	4	42	15
Koshima et al.2, 1985 (E)	15413	10120	4	13157	7829	3	49	15
Koshima et al.2, 1985 (F)	9001	2352	4	11422	11422	3	56	15
Koshima et al.2, 1985 (G)	14387	6512	6	9685	7418	3	84	15
Koshima et al.2, 1985 (H)	17457	4951	3	13276	3656	5	112	15
Koshima et al.2, 1985 (I)	14098	3059	4	19147	6639	3	140	15
Koshima et al.2, 1985 (J)	11819	1366	3	18629	3366	3	168	15
Koshima et al.2, 1985 (K)	14398	1306	3	-	-	-	224	15
Matsumine et al., 2013	7986	2794	7	5264	774	7	210	7
Ozcan et al., 1993	7666	1125	4	7529	2078	6	84	10
Seckel et al., 1986 (A)	4734	1804	9	5629	2034	9	21	10
Seckel et al., 1986 (B)	2490	15	2	2337	789	2	28	10
Seckel et al., 1986 (C)	4559	1460	2	3642	190	2	42	10
Shibata et al., 1988 (A)	365	723	11	744	909	11	70	30
Shibata et al., 1988 (B)	2207	1077	9	1411	2520	8	168	30
Tark et al., 2001 (A)	84	12	11	9	1	11	56	40
Tark et al., 2001 (B)	53	12	11	27	2	11	84	40
Tark et al., 2001 (C)	98	13	11	86	12	11	112	40

vng = vascularized nerve graft

cng = conventional nerve graft

Diameter

study name	vng mean	vng sd	vng n	cng mean	cng sd	cng n	days	graft size (mm)
Donzelli et al., 2016 (A)	3,5	0,3	5	3,1	0,5	5	30	
Donzelli et al., 2016 (B)	4,03	0,8	5	3,8	0,8	5	90	
Hems et al., 1992	1,99	-	4	1,77	-	4	250	50
Kawai et al., 1990 (A)	3,42	3,17	8	3,48	3,17	8	56	20
Kawai et al., 1990 (B)	4,6	5,21	5	3,68	3,26	6	56	40
Kawai, et al. 1990 (C)	4,73	3,6	3	4,03	2,3	3	56	60
Kawai et al., 1990 (D)	5,03	4,65	5	4,6	4,12	6	168	20
Kawai et al., 1990 (E)	5,36	7,35	6	4,72	3,99	6	168	40
Kawai et al., 1990 (F)	3,9	2,84	6	4,69	3,44	6	168	60
Koshima et al., 1985 (A)	0	0	4	0	0	4	28	15
Koshima et al., 1985 (B)	1,2	1,2	3	0	1,2	3	56	15
Koshima et al., 1985 (C)	2,8	0,4	4	2	0,2	4	84	15
Koshima et al., 1985 (D)	2,7	0,1	4	2,1	0,3	4	112	15
Koshima et al., 1985 (E)	4,2	0,3	2	2,5	0,3	2	140	15
Koshima et al., 1985 (F)	3,4	0,3	4	2,2	1,8	1	168	15
Koshima et al.2, 1985 (A)	0	0	3	0	0	4	21	15
Koshima et al.2, 1985 (B)	0,77	1,1	3	0	1,1	3	28	15
Koshima et al.2, 1985 (C)	0,6	0,85	3	0	0,85	4	35	15
Koshima et al.2, 1985 (D)	2,05	0,15	3	0,48	0,82	4	42	15
Koshima et al.2, 1985 (E)	1,87	0,21	4	1,8	0,21	3	49	15
Koshima et al.2, 1985 (F)	2,2	0,14	4	1,1	1,1	3	56	15
Koshima et al.2, 1985 (G)	2,63	0,53	6	0,85	1,2	3	84	15
Koshima et al.2, 1985 (H)	2,2	0,33	3	2,35	0,4	5	112	15
Koshima et al.2, 1985 (I)	2,6	0,3	4	1,9	0,14	3	140	15
Koshima et al.2, 1985 (J)	3,1	0,53	3	2,1	0,14	3	168	15
Koshima et al.2, 1985 (K)	2,7	0,36	3	-	-	-	224	15
Mani et al., 1992	5,03	0,36	6	5,72	1.01	5	308	30
Matsumine et al., 2013	7,69	2,75	3	5,28	2,08	3	210	7
Ozcan et al., 1993	4,92	0,27	4	3,89	0,37	6	84	10
Shibata et al., 1988 (A)	-	-	-	1,7	0	1	70	30
Shibata et al., 1988 (B)	2,4	0,73	6	2,8	0,85	2	168	30

vng = vascularized nerve graft

cng = conventional nerve graft

Nerve conduction velocity

study name	vng mean	vng sd	vng n	cng mean	cng sd	cng n	days	graft size (mm)
Kanaya et al., 1992	46,2	7,7	14	36,8	4,3	8	84	25
Mani et al., 1992	42	4,4	6	49	5,6	5	308	30
Shibata et al., 1988 (A)	24	6,3	10	20	3,2	10	70	30
Shibata et al., 1988 (B)	38	9,5	10	36	6,3	10	168	30
Zhu et al., 2015	33,1	0,6	6	30,2	0,8	6	112	20

vng = vascularized nerve graft

cng = conventional nerve graft

Muscle weight

study name	vng mean	vng sd	vng n	cng mean	cng sd	cng n	days	graft size (mm)
Bertelli et al., 1996 (A)	53,1	8,3	7	53,7	6,8	7	95	20
Bertelli et al., 1996 (B)	61	4	7	62,7	4,7	7	120	20
Bertelli et al., 1996 (C)	64,9	2	7	65,1	2,5	7	150	20
Bertelli et al., 1996 (D)	64,9	3,3	7	64,9	2,7	7	210	20
Bertelli et al., 1996 (E)	70,9	2,1	7	68,6	2,5	7	360	20
Kanaya et al., 1992	60	6,1	14	55,9	8	8	84	25

vng = vascularized nerve graft

cng = conventional nerve graft
