# Supplementary material for: Nerve recovery from treatment with a vascularized nerve graft compared to an autologous non-vascularized nerve graft in animal models: A systematic review and meta-analysis
Source: PLoS One. 2021 Dec 2;16(12):e0252250. doi: 10.1371/journal.pone.0252250 (PMC8638852; doi:10.1371/journal.pone.0252250)
Supplement: Attachment — Submitted filename: Respons to Reviewers .docx [file pone.0252250.s014.docx]

**Rebuttal letter**

PONE-D-21-15417
“Nerve recovery from treatment with a vascularized nerve graft compared to an autologous non-vascularized nerve graft in animal models: a systematic review and meta-analysis”

Berend O. Broeren^1*^, Liron S. Duraku^2^, Caroline A. Hundepool^2^, Erik T. Walbeehm^1^, J. Michiel Zuidam^2^, Carlijn R. Hooijmans^3,4^, Tim De Jong^1^

PLOS ONE

Leila Harhaus

Academic Editor

PLOS ONE

Dear Ms Harhaus,

We thank you and the reviewers for a careful reading and the constructive comments regarding our manuscript and for the opportunity to revise and resubmit. We have addressed all recommendations and suggestions to further improve the manuscript. On behalf of my co-authors, I thank you for considering this revised manuscript for publication. We appreciate your time and look forward to your response.

Yours sincerely,

Berend Broeren (corresponding author)

[berend.broeren@xs4all.nl](mailto:berend.broeren@xs4all.nl)

Journal requirements:

1. Please ensure that your manuscript meets PLOS ONE's style requirements, including those for file naming. The PLOS ONE style templates can be found at

<https://journals.plos.org/plosone/s/file?id=wjVg/PLOSOne_formatting_sample_main_body.pdf> and

<https://journals.plos.org/plosone/s/file?id=ba62/PLOSOne_formatting_sample_title_authors_affiliations.pdf>

2. In your Data Availability statement, you have not specified where the minimal data set underlying the results described in your manuscript can be found. PLOS defines a study's minimal data set as the underlying data used to reach the conclusions drawn in the manuscript and any additional data required to replicate the reported study findings in their entirety. All PLOS journals require that the minimal data set be made fully available. For more information about our data policy, please see <http://journals.plos.org/plosone/s/data-availability>.

Upon re-submitting your revised manuscript, please upload your study’s minimal underlying data set as either Supporting Information files or to a stable, public repository and include the relevant URLs, DOIs, or accession numbers within your revised cover letter. For a list of acceptable repositories, please see <http://journals.plos.org/plosone/s/data-availability#loc-recommended-repositories>. Any potentially identifying patient information must be fully anonymized.

Important: If there are ethical or legal restrictions to sharing your data publicly, please explain these restrictions in detail. Please see our guidelines for more information on what we consider unacceptable restrictions to publicly sharing data: <http://journals.plos.org/plosone/s/data-availability#loc-unacceptable-data-access-restrictions>. Note that it is not acceptable for the authors to be the sole named individuals responsible for ensuring data access.

We will update your Data Availability statement to reflect the information you provide in your cover letter.

**We have added our raw data using a supporting file and included a caption at the end of the manuscript.**

3. Please include a separate caption for each figure in your manuscript.

**We have included a caption for each figure immediately following the paragraph in which the figure was first cited with a label, title and legend.**

4. Please include captions for your Supporting Information files at the end of your manuscript, and update any in-text citations to match accordingly. Please see our Supporting Information guidelines for more information: [http://journals.plos.org/plosone/s/supporting-information](about:blank)

**We have included a caption for each supporting information file at the end of the manuscript with a label, title and legend. In-text citations were matched accordingly.**

Comments to the Author

1. Is the manuscript technically sound, and do the data support the conclusions?

The manuscript must describe a technically sound piece of scientific research with data that supports the conclusions. Experiments must have been conducted rigorously, with appropriate controls, replication, and sample sizes. The conclusions must be drawn appropriately based on the data presented.

Reviewer #1: Yes

Reviewer #2: Partly

2. Has the statistical analysis been performed appropriately and rigorously?

Reviewer #1: Yes

Reviewer #2: N/A

3. Have the authors made all data underlying the findings in their manuscript fully available?

The [PLOS Data policy](http://www.plosone.org/static/policies.action#sharing) requires authors to make all data underlying the findings described in their manuscript fully available without restriction, with rare exception (please refer to the Data Availability Statement in the manuscript PDF file). The data should be provided as part of the manuscript or its supporting information, or deposited to a public repository. For example, in addition to summary statistics, the data points behind means, medians and variance measures should be available. If there are restrictions on publicly sharing data—e.g. participant privacy or use of data from a third party—those must be specified.

Reviewer #1: Yes

Reviewer #2: Yes

4. Is the manuscript presented in an intelligible fashion and written in standard English?

PLOS ONE does not copyedit accepted manuscripts, so the language in submitted articles must be clear, correct, and unambiguous. Any typographical or grammatical errors should be corrected at revision, so please note any specific errors here.

Reviewer #1: Yes

Reviewer #2: Yes

5. Review Comments to the Author

Please use the space provided to explain your answers to the questions above. You may also include additional comments for the author, including concerns about dual publication, research ethics, or publication ethics. (Please upload your review as an attachment if it exceeds 20,000 characters)

Reviewer #1:

Nerve recovery from treatment with a vascularized nerve graft compared to an autologous non-vascularized nerve graft in animal models: a systematic review and meta-analysis
This manuscript is a metanalysis comparing vascularized vs. no vascularized nerve graft. The article fits the scope of the journal. The title appropriately summarizes the article contents. The article is well-structured, the language is adequate. The references are uniform and according to the instructions for authors. The main concern is the heterogenous data with poor methodological details from the different manuscripts this paper is based on.
Please consider the next suggestions/clarification to improve the manuscript:
- Axonal account and diameter: were a uniform histological technique regarding the axonal account? Paper’s years of publication range from 1988 until 2016. Although these histological techniques are not novel, the sensitivity and specific of this technique has been improved during the last 30 years. Do the authors think it could exist a bias comparing these studies?

**We appreciate the positive comments of Reviewer #1 and share the concerns regarding the heterogenous data and the poor methodological details from the different manuscripts this paper is based on. Therefore, it was extensively described in the section ‘limitations of this study” and the recommendation to use the Gold Standard Publication Checklist or ARRIVE guidelines for future animal studies to improve the reporting and methodological quality of animal studies in our conclusion was made. As to the uniformity of histological technique to measure axonal count, we added a section in the “limitations of this study” section. Different techniques were used by the studies in this review. To compensate for these differences, we used a standardized mean difference for our meta-analysis. The statement made by Reviewer #1 that these techniques have evolved is true and the sensitivity and specific of this technique may have been improved during the last 30 years. Over the years methods have evolved from manually calculating axonal count from a light microscopic photograph to a computer calculated estimate. Searching the publication databases, we found little evidence on which one is the best or on a clear sensitivity or specificity for these methods. Kim et al. concluded that semi-automated method for counting axons in transmission electron microscopic images were strongly correlated with those of conventional counting methods and showed excellent reproducibility. Nevertheless, the techniques for histomorphometry will always be an estimation and therefore prone to bias.**

- I have a similar concern regarding the three studies with neurophysiology. This technique needs an experience neurologist or neurophysiologist to get reliable data.

**The studies where neurophysiology was one of the outcome measurements did not mention the skillset of the person who assessed the outcomes. The data could possibly be biased but this cannot be established with the information at hand.**

I recommend the publication of the manuscript after the above comments are addressed. We thanks the authors the great effort needed for this study.

Reviewer #2:

What is the personal guidelines in your institution? Please add your guideline into this paper.

**Although we don’t have a clinical guideline for peripheral nerve gaps, we have added information of the clinical practice of our institution in the introduction of the manuscript.**

**The use of vascularized nerve grafts is debated in our medical centers because of the lack of clinical evidence indicating the superiority of a vascularized nerve graft, the concern of a more significant donor site morbidity compared to conventional nerve autografts and the complexity of the surgery.**

The figures are in poor resolution, blurred and not clear.
Kindly ask you improve the resolution.

**All figures have been improved using the PACE digital diagnostic tool to make sure the resolution improved and the PLOS requirements were met.**
