# Supplementary material for: Nerve recovery from treatment with a vascularized nerve graft compared to an autologous non-vascularized nerve graft in animal models: A systematic review and meta-analysis
Source: PLoS One. 2021 Dec 2;16(12):e0252250. doi: 10.1371/journal.pone.0252250 (PMC8638852; doi:10.1371/journal.pone.0252250)
Supplement: Attachment — Submitted filename: Respons to reviewers.docx [file pone.0252250.s015.docx]

**Rebuttal letter**

PONE-D-21-15417R1
“Nerve recovery from treatment with a vascularized nerve graft compared to an autologous non-vascularized nerve graft in animal models: a systematic review and meta-analysis”

Berend O. Broeren^1*^, Liron S. Duraku^2^, Caroline A. Hundepool^2^, Erik T. Walbeehm^1^, J. Michiel Zuidam^2^, Carlijn R. Hooijmans^3,4^, Tim De Jong^1^

PLOS ONE

Leila Harhaus

Academic Editor

PLOS ONE

Dear Ms Harhaus,

We thank you and the reviewer for a careful reading and the constructive comments regarding our manuscript and for the opportunity to revise and resubmit. We have addressed all recommendations and suggestions to further improve the manuscript. On behalf of my co-authors, I thank you for considering this revised manuscript for publication. We appreciate your time and look forward to your response.

Yours sincerely,

Berend Broeren (corresponding author)

[berend.broeren@xs4all.nl](mailto:berend.broeren@xs4all.nl)

Journal Requirements:

Please review your reference list to ensure that it is complete and correct. If you have cited papers that have been retracted, please include the rationale for doing so in the manuscript text, or remove these references and replace them with relevant current references. Any changes to the reference list should be mentioned in the rebuttal letter that accompanies your revised manuscript. If you need to cite a retracted article, indicate the article’s retracted status in the References list and also include a citation and full reference for the retraction notice.

**The reference list is complete and correct**

Comments to the Author

1. If the authors have adequately addressed your comments raised in a previous round of review and you feel that this manuscript is now acceptable for publication, you may indicate that here to bypass the “Comments to the Author” section, enter your conflict of interest statement in the “Confidential to Editor” section, and submit your "Accept" recommendation.

Reviewer #3: (No Response)

2. Is the manuscript technically sound, and do the data support the conclusions?

The manuscript must describe a technically sound piece of scientific research with data that supports the conclusions. Experiments must have been conducted rigorously, with appropriate controls, replication, and sample sizes. The conclusions must be drawn appropriately based on the data presented.

Reviewer #3: Partly

3. Has the statistical analysis been performed appropriately and rigorously?

Reviewer #3: Yes

4. Have the authors made all data underlying the findings in their manuscript fully available?

The [PLOS Data policy](http://www.plosone.org/static/policies.action#sharing) requires authors to make all data underlying the findings described in their manuscript fully available without restriction, with rare exception (please refer to the Data Availability Statement in the manuscript PDF file). The data should be provided as part of the manuscript or its supporting information, or deposited to a public repository. For example, in addition to summary statistics, the data points behind means, medians and variance measures should be available. If there are restrictions on publicly sharing data—e.g. participant privacy or use of data from a third party—those must be specified.

Reviewer #3: Yes

5. Is the manuscript presented in an intelligible fashion and written in standard English?

PLOS ONE does not copyedit accepted manuscripts, so the language in submitted articles must be clear, correct, and unambiguous. Any typographical or grammatical errors should be corrected at revision, so please note any specific errors here.

Reviewer #3: Yes

6. Review Comments to the Author

Please use the space provided to explain your answers to the questions above. You may also include additional comments for the author, including concerns about dual publication, research ethics, or publication ethics. (Please upload your review as an attachment if it exceeds 20,000 characters)

Reviewer #3: The study provided an interesting overview of the field of vascularized nerve grafts, indicating that vascularized nerve grafts can achieve superior results in terms of axon count, axon diameter, and nerve conduction velocity when compared to non-vascularized nerve grafts.

However, the manuscript needs some major and minor revision before publication.

Major Points

The introduction should include a more detailed explanation of the technique and rationale behind these techniques. The author discussed the disadvantages of the vascularized nerve graft but did not discuss its advantages over the conventional ANT.

**We have added information on the technique and rationale behind the technique in the introduction of the manuscript. Page 4 line 72-74 and 76-80**

Interestingly, the author emphasizes the critical nature of functional recovery but includes no data in the manuscript, implying that vascularized nerve grafts may have an effect on functional recovery. While we are well aware of the heterogeneity associated with assessing functional recovery following peripheral nerve injury, these issues should also be discussed in the manuscript.

**We have added information on the rationale behind the choice of our outcome measurements and discuss the relevance of functional outcomes. Page 16 line 350, 351**

This manuscript provided compelling evidence that a peripheral nerve surgeon should place a greater emphasis on the vascularized nerve graft. The author, however, did not discuss the findings under "Discussion."

**The findings were indeed discussed in the “Discussion”.**

**This review suggests that a vascularized nerve graft does result in a significantly better nerve recovery compared to non-vascularized nerve autografts in animal models regarding the outcome measurements nerve fiber diameter, nerve conduction velocity and axonal count**. **Page 15 line 326-328**

**Treating a nerve gap with a vascularized graft results in superior nerve recovery compared to non-vascularized autografts nerve grafts in three out of four outcome measurements. Page 19 line 414, 415**

What is the author's opinion about the superior axon count and diameter? Why do you see the effects even for the nerve conduction velocity and not for the muscle weight?

**We have added information on how the authors think about the superior axon count and diameter and the lacking effect in muscle weight. See page 16 line 334-339**

Justifies the advantages of an enlarged donor site the disadvantages?

**We have added information on how the authors think about the question if the advantages of a vascularized nerve graft justify the disadvantages of an enlarged donor site. Page 17 line 366-370**

Can the findings of an animal study be applied to humans?

**We think this is a fair question. However, we also think this cannot be said with certainty. Animal research comes closed to clinical research and is therefore the most appropriate alternative if clinical research is not available. Of course, something can be said for the fact that research in primates comes closer to humans than, say, the rat. In particular when it comes to nerve grafts because of the length and diameter of the nerve graft. Therefore, we recommended future studies to be performed under conditions more closely resembling human circumstances and to use long nerve grafts.**

Therefore the Discussion section should be appropriately revised.

Minor Points

Abstract


Line 54: What does this sentence mean? Three out of four measurements. In which measurements is a vascularized nerve graft superior?

**This sentence was changed to clarify it. See page 3 line 54, 55**

Introduction:

The author should give a short overview of the technique of the vascularized nerve graft and explain its rationality further –

**Changed, page 4 line 72-74 and 76-80**

Line 63: When is it necessary to bridge the gap – when a tensionless coaptation is not possible – this should be added

**This had been added see page 3 line 64, 65**

Line 67: please rephrase the sentence – the meaning is clear – but the expression has to be improved

**This sentence has been rephrased see page 3 line 66-69**

Line 80: what did these studies show – please elaborate on the findings of the previous research

**Information has been added see page 4 line 82, 83**

Line 88: why do you use them and not nerve tubes? When you mean Axogen, then due to the results of the ranger study – these should be added.

**Did you mean line 98? Why we use autografts in most cases and not allografts? Autografts give a better outcome, with the down side of donor site morbidity which is minimal for most used nerve grafts. If not enough length of autografts is present, we use axogen.**

**We added references. See page 5 line 98, 99**

Line 117: what about functional regeneration? Did the studies give no information focusing on the functional regeneration compared to a "conventional" ANT

**No functional outcomes were described in more than one of the included articles. Only one described grasping strength and another one the sciatic function index.**

Material and Methods:

Figure 1: Can you please clarify why you exclude the 175 manuscripts to classify the arguments. Would you please adjust the figure?

**Figure was adjusted accordingly.**

Results:

Line 205; the meaning of the sentence is not clear – please rephrase

**Rephrased see page 10 line 215**

Line 210: please add the level of significance

**The significance was added, see page 11 line 226**

Line 235: why here SEM and not SMD?

**Changed to SMD, see page 12 line 250, 251**

Discussion:

This is a critical point in line 319 – 329. However, no data on functional recovery were provided, even with a small number of studies – despite the fact that this is the most important "test" for peripheral nerve injury recovery due to its clinical relevance.

**We rephrased this sentence. See page 16 line 350, 351**

Line 371: Because histomorphometry is crucial to this review, the author should explain how the axonal count was obtained. Counting axons using random field of interest methods and counting the entire diameter have varying degrees of validity for the data.

**We have added information on how axonal count was obtained in the Results of the manuscript. See page 11 line 220-224**
